# Inequalities in survival and care across social determinants of health in a cohort of advanced lung cancer patients in Quebec (Canada): A high‐resolution population‐level analysis

**DOI:** 10.1002/cam4.5897

**Published:** 2023-04-05

**Authors:** Samia Qureshi, Gino Boily, Jim Boulanger, Élisabeth Pagé, Erin Strumpf

**Affiliations:** ^1^ Department of Epidemiology, Biostatistics and Occupational Health (EBOH) McGill University H3A 1G1 Montreal, Quebec Canada; ^2^ Institut national d'excellence en santé et services sociaux (INESSS) H3A 2S9 Montreal, Quebec Canada; ^3^ Institut national d'excellence en santé et services sociaux (INESSS) H3A 2S9 Quebec, Quebec Canada

**Keywords:** health inequalities, lung neoplasms, molecular targeted therapy, sociodemographic factors, socioeconomic factors, survival analysis, time‐to‐treatment

## Abstract

**Background:**

Advanced lung cancer patients exposed to breakthrough therapies like EGFR tyrosine kinase inhibitors (EGFR‐TKI) may experience social inequalities in survival, partly from differences in care. This study examined survival by neighborhood‐level socioeconomic and sociodemographic status, and geographical location of advanced lung cancer patients who received gefitinib, an EGFR‐TKI, as first‐line palliative treatment. Differences in the use and delay of EGFR‐TKI treatment were also examined.

**Methods:**

Lung cancer patients receiving gefitinib from 2001 to 2019 were identified from Quebec's health administrative databases. Accounting for age and sex, estimates were obtained for the median survival time from treatment to death, the probability of receiving osimertinib as a second EGFR‐TKI, and the median time from biopsy to receiving first‐line gefitinib.

**Results:**

Among 457 patients who received first‐line treatment with gefitinib, those living in the most materially deprived areas had the shortest median survival time (ratio, high vs. low deprivation: 0.69; 95% CI: 0.47–1.04). The probability of receiving osimertinib as a second EGFR‐TKI was highest for patients from immigrant‐dense areas (ratio, high vs. lowdensity: 1.95; 95% CI: 1.26–3.36) or from Montreal (ratio, other urban areas vs. Montreal: 0.39; 95% CI: 0.16–0.71). The median wait time for gefitinib was 1.27 times longer in regions with health centers peripheral to large centers in Quebec or Montreal in comparison to regions with university‐affiliated centers (95% CI: 1.09–1.54; *n* = 353).

**Conclusion:**

This study shows that real‐world variations in survival and treatment exist among advanced lung cancer patients in the era of breakthrough therapies and that future research on inequalities should also focus on this population.

## 1 INTRODUCTION

Lung cancer remains the deadliest cancer in Canada.^1^ Roughly, half of all lung cancer patients are diagnosed at stage IV, when the 1‐year survival probability is as little as 17%.^1^, ^2^ With the gradual introduction of breakthrough palliative treatments like precision medicine (i.e., targeted therapies in 2010 and immunotherapy in 2015),^3^, ^4^ survival trends are expected to rise in the overall and advanced (i.e., locally advanced or metastatic) lung cancer populations.^5^, ^6^ However, survival improvements in socioeconomically, sociodemographically, or geographically disadvantaged subpopulations may not necessarily occur at the same pace as in advantaged subpopulations. Despite a universal healthcare system providing free health services at the point of care for medically necessary treatments in all provinces, there is growing evidence of social inequalities in outcomes along the cancer control continuum in Canada.^7^

Due to suboptimal care, survival from different cancers, including lung cancer, may be shorter in socioeconomically, sociodemographically, or geographically disadvantaged groups.^8^, ^9^, ^10^, ^11^ For example, cancer patients with low socioeconomic status (SES), immigrant status, or living in specific regions in Canada are less likely to receive oncology consultations, surgery, radiotherapy, chemotherapy, and supportive care near death.^12^, ^13^, ^14^, ^15^ Cancer treatment delays can also impact survival,^16^ including delays for targeted treatment in advanced lung cancer.^17^ However, the evidence for SES‐ and geography‐based differences in timely treatment for lung cancer in Canada and elsewhere is mixed.^14^, ^18^, ^19^, ^20^, ^21^

There are very few lung cancer studies in Canada that focus on inequalities in survival by SES measures other than income or by sociodemographic status (SDS) (e.g., immigration), and on inequalities in palliative care with systemic treatments.^1^, ^13^ Also, income‐based inequalities in survival are not evident for Canadian patients diagnosed with stage IV lung cancer in 2010–2011,^1^ a period when precision medicine barely existed. Decision‐makers in Quebec are increasingly interested in the real‐world effectiveness of breakthrough palliative therapies for advanced lung cancer, like EGFR tyrosine kinase inhibitors (EGFR‐TKI). While the survival of EGFR‐TKI users in Quebec has been investigated,^22^, ^23^ inequalities in survival and treatments by patient subgroups remain unknown. Investigating these inequalities in Quebec is relevant, since Quebec accounts for 23% of the Canadian population^24^ and about 16% of all Canadian immigrants in Canada.^25^ Among the most populous provinces (Ontario, Quebec, British Columbia, and Alberta), it has the lowest median household income (Quebec vs. others in 2020 constant Canadian dollars: $53,800 vs. $63,000–$75,300)^26^ and a higher percentage of individuals with educational attainment below upper secondary levels (Quebec vs. others: 12% vs. 7%–8%).^27^ Studies of advanced lung cancer patients in the United States suggest that there may be SES‐based inequalities in survival,^28^ in the use of breakthrough palliative treatments,^29^ and geography‐based inequalities in the delay to initiate EGFR‐TKI treatment.^30^

This population‐based retrospective study sought to fill a knowledge gap on survival inequalities and potential drivers of these inequalities across understudied characteristics of advanced lung cancer patients living in the current context of breakthrough therapies in Canada. Among patients who received the first‐generation of EGFR‐TKIs, gefitinib, as first‐line palliative treatment in Quebec, we examined fluctuations in survival by income, education, material deprivation index, immigration, and geographic region, as well as variations in the use and delay of EGFR‐TKI treatment (i.e., use of a second EGFR‐TKI, osimertinib, and delay between the tumor biopsy and first EGFR‐TKI). Results from this study may also be relevant to other countries with similar socio‐economic contexts, such as developed or high‐income countries (i.e., United States, United Kingdom, European Union, and Australia) that have social inequalities in lung cancer treatment and survival,^9^, ^10^, ^12^, ^19^, ^20^ and countries with universal healthcare systems which include coverage of oral cancer medicines.^31^

## 2 MATERIALS AND METHODS

### 2.1 Study design and data

We conducted a population‐level retrospective study by selecting patients in Quebec whose first indication of lung cancer was between April 1, 2001 and March 31, 2019, and who received gefitinib as first‐line palliative treatment in that period. Data came from health administrative databases managed by the Régie de l'assurance maladie du Québec (RAMQ), which capture information related to public insurance plans for health and prescription drugs, and by the Ministère de la Santé et des Services sociaux (MSSS), that capture inpatient and outpatient health care utilization by the Quebec population. Gefitinib is only reimbursed in Quebec to treat advanced lung cancer patients who are naïve to systemic palliative treatment. Focusing on this treatment thus allowed us to identify advanced lung cancer patients that are close in time to their diagnosis date.

The dates for the first indication of lung cancer and the initial lung cancer diagnosis were determined based on previously established methods.^32^ Patients who filled a prescription for gefitinib at least once between April 1, 2001 and March 31, 2019 were identified in the community pharmacy services database through previously selected international nonproprietary names (INN) and drug identification numbers (DIN).^22^, ^23^ To form cohort 1 for survival analyses and study the use of osimertinib as a second EGFR‐TKI, we identified the line of treatment associated with patients' first gefitinib treatment using a verified algorithm^22^, ^23^ and excluded all patients who did not receive gefitinib as a first‐line palliative treatment. From this cohort (cohort 1), a separate cohort (cohort 2) was formed to study the time delay from receiving a tumor biopsy (or biopsy‐related intervention) to receiving treatment. To form cohort 2, we excluded patients who did not receive a biopsy or received it more than 6 weeks before their lung cancer diagnosis and/or more than 6 months before their first gefitinib treatment. The date of death was captured with the health insurance registry (FIPA).

This study was part of a Health System Impact Fellowship of the Canadian Institutes of Health Research and was conducted at the Institut national d'excellence en santé et services sociaux (INESSS). De‐identified data was accessible through a tripartite agreement between MSSS, RAMQ, and INESSS.^33^ INESSS is not responsible for the content of this publication, however, the cohort used in this study is also included in a larger study by INESSS on EGFR‐TKIs in Quebec.^22^, ^23^

### 2.2 Outcomes

Survival time was calculated in months from the date of first gefitinib prescription to either the date of death or March 31, 2020: the administrative censoring time. Previously selected INNs and DINs were used to identify patients who received osimertinib as a second EGFR‐TKI during their follow‐up after receiving gefitinib.^22^, ^23^ Medical interventions involving biopsies that occurred between patients' diagnosis date^32^ and their first gefitinib treatment were extracted from physician billing and hospitalization databases with expert‐validated billing and intervention codes (Appendix A).^34^, ^35^ Treatment delays were calculated as the number of days between each patient's most recent biopsy and their first gefitinib treatment.

### 2.3 Exposures and covariates

FIPA was consulted for patients' age, sex, and areas of residence (1: the census‐based dissemination area [DA], which are the smallest standard geographic unit [400–700 persons] at the national level; and 2: one of 18 health regions) at the time of their first gefitinib treatment. Patients' DAs were linked to neighborhood‐level SES and SDS, and geography variables developed by INESSS with the 2016 census data and the overall RAMQ population in that year. The DA‐level SES and SDS variables included categorical information on median income after tax, percent of low education, and percent of individuals who immigrated to Canada. The census‐based geography variable classified patients' DA based on urbanicity: (1) DAs in Census Metropolitan Areas (CMA), which include Montreal and other large cities, were urban; (2) DAs in Census Agglomerations (CA) were suburban; and (3) all other DAs (non‐CMAs/CAs) were rural. Montreal was analyzed separately from other CMAs since it includes 86% of all immigrants in Quebec.^36^ Patients DAs were also linked to Pampalon's material deprivation index, which was developed with Quebec's 2016 Census population and is a composite score of income, education, and employment measures at the DA‐level that have been standardized to Quebec's age and sex distributions.^37^ Another geography variable we used, that is health center‐based, delineated patients' health regions according to the presence of university‐affiliated medical centers and the distance of other health centers from large health services centers in Montreal and Quebec.^38^, ^39^ More details on each exposure are listed in Table B1 (Appendix B). Comorbidities were calculated with the Population Grouping Methodology,^40^, ^41^ which uses diagnostic codes to search for 226 health conditions (excluding lung cancer) in the 3 years leading up to the date of the first gefitinib treatment.

### 2.4 Statistical analyses

Medians and proportions were calculated for patients' baseline characteristics. The median follow‐up time in cohort 1 was estimated with the distribution of censoring times (reverse Kaplan–Meier method) with the *prodlim* package in R.^42^ We used the *survival* package in R to conduct inverse‐probability‐of‐treatment‐weighted (IPTW) Kaplan–Meier analyses.^43^ Stabilized weights for IPTW were calculated with predicted treatment probabilities obtained from multinomial logistic regressions. The weighing produced a pseudo‐population with age and sex distributions at each category of SES, SDS, and geography that are like those in the overall unweighted population. For each exposure level, survival curves were plotted. The ratios of median overall survival (OS) times were also estimated from the Kaplan–Meier analyses, and 95% confidence intervals were obtained with a stratified case resampling method for right‐censored data.^44^

Osimertinib, a third‐generation EGFR‐TKI, can increase overall survival when prescribed in place of chemotherapy as a subsequent line after another EGFR‐TKI.^45^ We set out to identify variations in the use of this treatment, which might explain potential survival inequalities observed in the same cohort. Therefore, we ran age‐ and sex‐adjusted quasi‐Poisson regressions to estimate the ratios of marginal probabilities of receiving osimertinib as a second EGFR‐TKI treatment across SES, SDS, and geography in cohort 1. Bootstrapping was used to obtain 95% confidence intervals. Osimertinib was first approved for coverage by Quebec's public drug insurance program in November 2018. We assumed that the fraction of patients in our study that died before this period, who could not receive osimertinib, was non‐differential across SES, SDS, and geography. The validity of this assumption was tested in a secondary analysis that included only patients who were alive after October 31, 2018.

In cohort 2, we estimated the 25th, 50th, and 75th percentiles of treatment delays as the shorter, median, and longer delays, respectively, in each category of SES, SDS, and geography. The weighting method applied to cohort 1 was also used for this analysis. At each percentile (25th, 50th, or 75th), we estimated ratios of delays across categories of SES, SDS, and geography, and obtained bootstrapped 95% confidence intervals.

With an expected small sample, our exploratory analyses did not include statistical significance testing. We interpreted our results based on the direction and magnitude of the point estimates and the width of the associated confidence intervals.

## 3 RESULTS

### 3.1 Cohort characteristics

**FIGURE 1:**
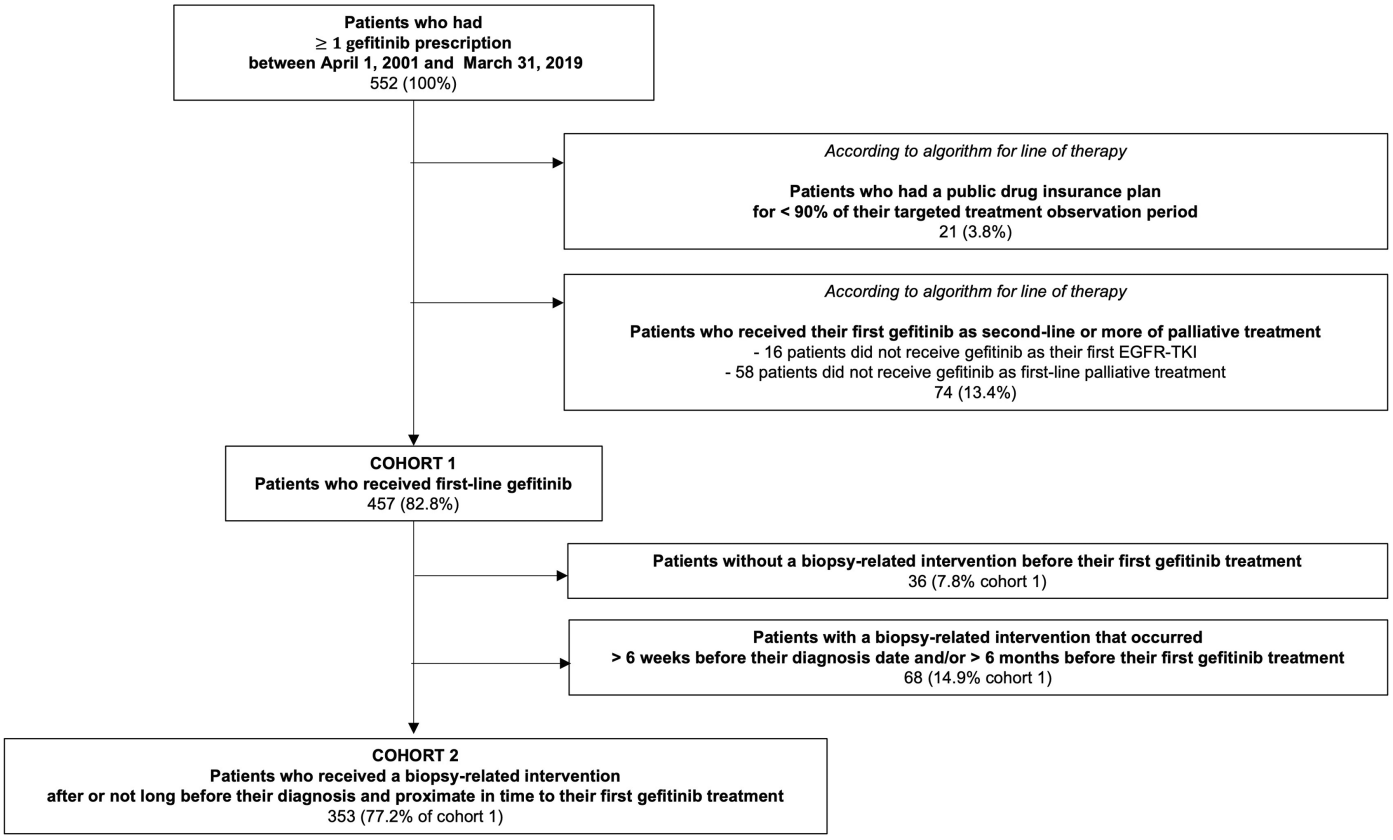
Flowchart of study population in each cohort.

Of the 552 patients who received gefitinib between April 1, 2001 and March 31, 2019, 457 patients (82.8%) received gefitinib as a first‐line palliative treatment, so they were included in cohort 1. Of the 457 patients, 353 (77.2%) patients were included in cohort 2 (Figure 1). Only 1 patient received their first gefitinib before the fiscal year 2011, and more than half of all patients received it in 2015 or afterward, both in cohort 1^23^ and cohort 2. For patients in cohort 1, the median age at first gefitinib treatment was 70 years, and the median time to treatment from diagnosis was 3 months (IQR: 1.9–12.9 months) (Table 1). A large percentage of patients were female (68.5%) or did not receive osimertinib as a second EGFR‐TKI treatment (79.9%). Most patients had between 0 and 9 comorbidities (73%), of which half had less than 5 comorbidities (36.5%). A higher proportion of patients resided in areas with the lowest income quintile (26.0%), or the highest quintiles of low education (25.4%) or immigration density (31.9%). At least half of all patients resided in Montreal (55.6%) or in areas with university‐affiliated health centers (51.0%). There were no major differences in cohort 2 in comparison to cohort 1, except for a slightly shorter time from diagnosis to treatment (median: 2.6 months, IQR: 1.8–4.9 months). Because of the small sizes of the middle categories of income, education, and immigration variables, and the suburban category of the census‐based region variable, we refrained from interpreting any comparisons made with these categories.

**TABLE 1:** Patient characteristics (unweighted populations).

	Cohort #1 *n* (%)	Cohort #2 *n* (%)
Age in years		
<60	58 (12.7)	51 (14.4)
60–64	48 (10.5)	43 (12.2)
65–70	102 (22.3)	82 (23.2)
≥70	249 (54.5)	177 (50.1)
Median (IQR)	70.6 (65.4–77.6)	70.0 (64.2–77.1)
Female	313 (68.5%)	252 (71.3%)
Comorbidities		
0–4	169 (37.0)	129 (36.5)
5–9	160 (35.0)	129 (36.5)
≥10	127 (27.7)	94 (26.6)
Missing	1 (0.2)	1 (0.28)
Median (IQR)	6 (3–10)	6 (3–10)
Osimertinib as second EGFR‐TKI Treatment	92 (20.1)	73 (20.7)
First osimertinib in 2016	10 (2.2)	10 (2.8)
First osimertinib in 2017	20 (4.4)	14 (4.0)
First osimertinib in 2018	42 (9.2)	34 (9.6)
First osimertinib in 2019	20 (4.4)	15 (4.2)
Months between diagnosis and first gefitinib treatment Median (IQR)	3 (1.9–12.9)	2.6 (1.8–4.9)
Median income after tax (quintiles)		
Low (quintiles 1 and 2): 0–28,222$	200 (43.7)	150 (42.4)
Medium (quintile 3): 28,223$–31,653$	99 (21.7)	76 (21.5)
High (quintiles 4 and 5): ≥31,654$	151 (33.1)	123 (34.9)
Missing	7 (1.5)	7 (1.1)
Percent with low education (quintiles)		
Low (quintiles 1 and 2): 0%–28.69%	206 (45.1)	166 (46.9)
Medium (quintile 3): 28.7%–34.99%	77 (16.8)	54 (15.3)
High (quintiles 4 and 5): ≥35.00%	171 (37.5)	132 (37.4)
Missing	3 (0.66)	3 (0.28)
Material deprivation index (terciles)		
Low (tercile 1: least deprived)	147 (32.2)	117 (33.1)
Medium (tercile 2)	126 (27.6)	99 (28.0)
High (tercile 3: most deprived)	152 (33.3)	114 (32.3)
Missing	32 (7.0)	23 (6.5)
Percent immigrant (quintiles)		
Low (quintiles 1 and 2): 0%–4.09%	152 (33.3)	114 (32.4)
Medium (quintile 3): 4.10%–10.29%	70 (15.3)	57 (16.1)
High (quintiles 4 and 5): ≥10.30%	232 (50.7)	71 (51.3)
Missing	3 (0.66)	1 (0.28)
Census‐based region		
Urban: Montreal	254 (55.6)	194 (55.0)
Urban: Others	83 (18.2)	65 (18.4)
Suburban	41 (9.0)	33 (9.3)
Rural	77 (16.8)	60 (16.9)
Missing	2 (0.44)	1 (0.28)
Health center‐based region		
University	233 (51.0)	178 (50.4)
Peripheral	142 (31.1)	116 (32.9)
Intermediary	64 (14.0)	48 (13.6)
Remote or Northern	16 (3.5)	10 (2.8)
Missing	2 (4.4)	1 (0.28)
Total (*N*)	457	353

Abbreviations: IQR: interquartile range; SD, standard deviation.

### 3.2 Overall survival

**FIGURE 2:**
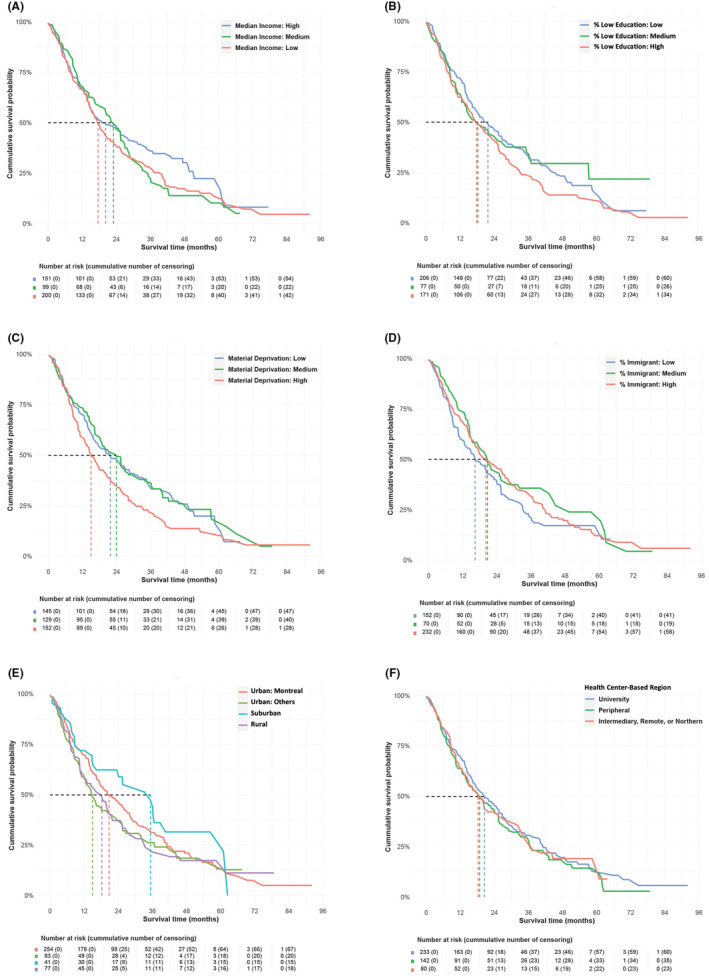
Overall survival with gefitinib as first‐line treatment, by socioeconomic, sociodemographic, and geographic factors (age‐ and sex‐standardized, weighted populations).

With a total of 337 deaths, the median follow‐up and overall survival times were, respectively, 45.3 months (IQR: 27.3–57.1) and 19.8 months (95% CI: 17.1–23.1 months). Our application of IPTW was successful in balancing age and sex distributions across categories of SES, SDS, and geographic location (Appendix C). Age‐ and sex‐standardized survival patterns across SES, SDS, and geographic location in the weighted populations are presented in Figure 2. The ratios in median survival times in Table 2 that were obtained from the inverse probability‐weighted survival curves show that patients from neighborhoods with the highest percentage of low education had a shorter survival time (high vs. low: 0.82; 95% CI: 0.64–1.17), as did those living in areas with the highest level of material deprivation index (high vs. low: 0.69; 95% CI: 0.47–1.04). In contrast, patients from neighborhoods with the highest immigrant density had a longer survival time (ratio, high vs. low: 1.23; 95% CI: 0.84–1.73). Patients living in urban areas other than Montreal had a lower median survival time than those living in Montreal (ratio: 0.71; 95% CI: 0.52–1.04). The ratios of median survival times by income groups and health center‐based regions were the smallest.

**TABLE 2:** Median overall survival (age‐ and sex‐standardized, weighted populations).

	Median survival time^a^ months (95% CI)	Median survival time ratio^a^ (95% CI)
SES and SDS variables		
Income		
Low: 0–28,222$	17.6 (15.1–21.1)	0.87 (0.60–1.26)
Medium: 28,223$–31,653$	23.0 (16.7–27.0)	1.14 (0.71–1.66)
High: ≥31,654$	20.2 (14.6–28.0)	REF
Low Education		
Low: 0%–28.69%	21.7 (17.3–25.6)	REF
Medium: 28.7%–34.99%	18.3 (12.6–28.3)	0.84 (0.56–1.49)
High: ≥35.00%	17.8 (15.0–23.0)	0.82 (0.64–1.17)
Material deprivation index		
Low (least deprived)	21.7 (16.4–29.6)	REF
Medium	23.8 (18.1–29.4)	1.10 (0.70–1.59)
High (most deprived)	15.1 (11.8–18.2)	0.69 (0.47–1.04)
Immigration		
Low: 0%–4.09%	16.4 (12.6–22.9)	REF
Medium: 4.10%–10.29%	20.7 (14.6–28.3)	1.26 (0.77–1.93)
High: ≥10.30%	20.1 (16.3–25.9)	1.23 (0.84–1.73)
Geography variables		
Census‐based region		
Urban: Montreal	20.8 (17.6–25.9)	REF
Urban: Others	14.9 (11.9–21.7)	0.71 (0.52–1.04)
Suburban	35.3 (15.1–40.6)	1.70 (0.69–2.16)
Rural	18.2 (11.1–21.9)	0.87 (0.51–1.16)
Health center‐based region		
University	20.5 (16.7–25.5)	REF
Peripheral	18.8 (14.3–24.1)	0.92 (0.63–1.31)
Intermediary, remote or northern	18.3 (13.1–32.1)	0.89 (0.57–1.58)

Abbreviations: SDS, Sociodemographic status; SES, Socioeconomic status.

^a^ Analyses accounted for age (<60, 60–64, 65–70, ≥70 years) and sex.

### 3.3 Probability of receiving osimertinib as a second EGFR‐TKI treatment

Estimates in Table 3 show that patients living in neighborhoods with the highest levels of low education, or material deprivation index, in comparison to the lowest levels, had lower probabilities of receiving osimertinib as a second EGFR‐TKI treatment (ratio and 95% CI, respectively: 0.69, 0.43–1.11; and 0.75, 0.43–1.22). Patients from the highest immigrant‐dense areas were twice as likely to receive osimertinib (ratio, high vs. low: 1.95; 95% CI: 1.26–3.36). Conversely, patients living in other urban areas or rural areas had much lower probabilities of receiving osimertinib compared to patients from Montreal (ratio and 95% CI, respectively: 0.39, 0.16–0.71; and 0.55, 0.25–0.98). The ratios across income and health center‐based regions were the smallest.

**TABLE 3:** Receiving osimertinib as a second EGFR‐TKI after receiving gefitinib (age and sex‐standardized, unweighted populations).

	Percentage^a^ (95% CI)	Percentage ratio^a^ (95% CI)
SES and SDS variables		
Income		
Low: 0–28,222$	17.8 (12.5–22.6)	0.85 (0.54–1.33)
Medium: 28,223$–31,653$	22.2 (14.4–30.4)	1.05 (0.62–1.72)
High: ≥31,654$	21.5 (14.7–28.8)	REF
Low Education		
Low: 0%–28.69%	24.3 (18.6–29.5)	REF
Medium: 28.7%–34.99%	15.8 (7.6–24.2)	0.65 (0.31–1.14)
High: ≥35.00%	16.9 (11.1–22.8)	0.69 (0.43–1.11)
Material deprivation index		
Low (least deprived)	21.7 (15.6–29.1)	REF
Medium	24.2 (16.8–32.9)	1.12 (0.69–1.82)
High (most deprived)	16.3 (10.3–22.2)	0.75 (0.43–1.22)
Immigration		
Low: 0%–4.09%	12.7 (7.4–18.1)	REF
Medium: 4.10%–10.29%	19.8 (11.0–28.8)	1.56 (0.75–3.0)
High: ≥10.30%	24.8 (19.4–29.9)	1.95 (1.26–3.36)
Geography variables		
Census‐based region		
Urban: Montreal	24.1 (18.5–29.6)	REF
Urban: Others	9.4 (3.8–16.1)	0.39 (0.16–0.71)
Suburban	28.9 (14.7–44.5)	1.20 (0.60–2.05)
Rural	13.3 (5.9–22.1)	0.55 (0.25–0.98)
Health center‐based region		
University	19.8 (14.4–25.4)	REF
Peripheral	22.7 (15.3–29.0)	1.15 (0.74–1.71)
Intermediary, remote or northern	16.1 (8.1–24.9)	0.81 (0.42–1.38)

Abbreviations: SDS, sociodemographic status; SES, socioeconomic status.

^a^ Analyses accounted for age (<60, 60–64, 65–70, ≥70 years) and sex.

### 3.4 Treatment delays

The overall median time between a biopsy and first gefitinib treatment was 39 days (IQR: 26–55). The largest ratios of treatment delays were observed by geographical location and across all quantiles of treatment delays in the weighted populations (Table 4). Patients living in other urban areas waited shorter than those in Montreal (delay ratios and 95% CIs of 25th, 50th, and 75th percentiles, respectively: 0.70, 0.56–1.07; 0.79, 0.66–0.95; and 0.83, 0.63–1.00). In contrast, patients living in peripheral regions waited longer than those in university regions (delay ratios and 95% CIs of 25th, 50th, and 75th percentiles, respectively: 1.48, 1.18–2.07; 1.27, 1.09–1.54; and 1.29, 1.11–1.48).

**TABLE 4:** Delay in receiving first‐line treatment after receiving a biopsy‐related intervention (age and sex‐standardized, weighted populations).

	25th percentile		50th percentile		75th percentile	
	Delay in days^a^	Delay ratio^a^	Delay in days^a^	Delay ratio^a^	Delay in days^a^	Delay ratio^a^
	(95% CI)	(95% CI)	(95% CI)	(95% CI)	(95% CI)	(95% CI)
SES and SDS variables						
Income						
Low: 0–28,222$	25.3 (20.4–31.9)	1.05 (0.77–1.49)	39 (36–42)	1.08 (0.86–1.21)	52 (47–59.9)	0.90 (0.76–1.09)
Medium: 28,223$–31,653$	27.8 (22.7–32.7)	1.15 (0.91–1.45)	37.5 (32.6–42)	1.04 (0.82–1.23)	51.0 (43–57)	0.89 (0.73–1.08)
High: ≥31,654$	24 (19.7–29.4)	REF	36 (33–42.2)	REF	57.5 (49.7–65)	REF
Low Education						
Low: 0%–28.69%	23.2 (19.6–27.1)	REF	36 (33.5–40.9)	REF	55 (48.8–60.4)	REF
Medium: 28.7%–34.99%	30 (25.7–36)	1.29 (1.06–1.74)	40.5 (35.8–45)	1.13 (0.96–1.27)	54.6 (45–64.8)	0.99 (0.80–1.17)
High: ≥35.00%	28 (22–33)	1.21 (0.96–1.58)	40 (35–42)	1.11 (0.93–1.22)	54.2 (48–61.9)	0.99 (0.86–1.15)
Material deprivation index						
Low (least deprived)	21 (19.0–28.8)	REF	36 (33–45.8)	REF	57 (50–66)	REF
Medium	28.8 (27–32.2)	1.37 (1.00–1.58)	39 (35–44)	1.08 (0.88–1.25)	52 (46–59)	0.91 (0.75–1.08)
High (most deprived)	26.2 (21.8–32.8)	1.25 (0.87–1.57)	39 (35–42)	1.08 (0.85–1.22)	51.5 (45–58)	0.90 (0.76–1.06)
Immigration						
Low: 0%–4.09%	26 (21–28)	REF	35 (30–40)	REF	48.7 (42–57.5)	REF
Medium: 4.10%–10.29%	29.0 (21–36.1)	1.11 (0.80–1.49)	40 (35–47.3)	1.14 (0.92–1.41)	55.9 (49–73.8)	1.15 (0.91–1.55)
High: ≥10.30%	25.0 (20.0–31)	0.96 (0.77–1.27)	39 (36–43)	1.11 (0.92–1.32)	56.0 (50.0–62.4)	1.15 (0.93–1.36)
Geography variables						
Census‐based region						
Urban: Montreal	30 (23.0–33)	REF	42 (38–46.8)	REF	58 (53.9–64.2)	REF
Urban: Others	21 (17–29.9)	0.70 (0.56–1.07)	33 (29.2–37.9)	0.79 (0.66–0.95)	48 (37–58)	0.83 (0.63–1.00)
Suburban	27.0 (21.9–34.0)	0.90 (0.71–1.26)	38.8 (28–45)	0.92 (0.63–1.08)	48.9 (40–88)	0.84 (0.66–1.53)
Rural	23.4 (21–29)	0.78 (0.66–1.13)	35 (27.8–41)	0.83 (0.65–1.02)	52.2 (40.6–67)	0.90 (0.67–1.13)
Health center‐based region						
University	21 (15.5–25)	REF	35 (31–37.6)	REF	48 (44.6–53.3)	REF
Peripheral	31 (27.0–35)	1.48 (1.18–2.07)	44.5 (40–54)	1.27 (1.09–1.54)	62 (56.9–70)	1.29 (1.11–1.48)
Intermediary, remote or northern	27.3 (22.2–32.8)	1.30 (1.03–1.81)	39 (30.5–42)	1.11 (0.86–1.29)	51.9 (42–65)	1.08 (0.81–1.34)

Abbreviations: SDS, sociodemographic status; SES, socioeconomic status.

^a^ Analyses accounted for age (<60, 60–64, 65–70, ≥70 years) and sex.

## 4 DISCUSSION

Using population‐based health‐administrative data, we investigated whether inequalities in survival and treatment services exist among advanced lung cancer patients receiving gefitinib as first‐line palliative treatment in Quebec. We observed shorter survival among patients residing in areas most representative of low education, high material deprivation, and minimal immigrant density in relation to the least representative areas. Patients from rural areas, and from urban areas other than Montreal, had similarly shorter median survival times than Montreal patients.

Our conclusions are qualitatively like several studies in Canada and elsewhere that investigated inequalities in survival from lung cancer by income, immigration status, and rurality in stage non‐specific populations.^14^, ^28^, ^46^, ^47^, ^48^, ^49^, ^50^, ^51^, ^52^, ^53^ Two Canadian studies report no associations of survival with income in advanced lung cancer patients or with rurality in lung cancer patients of whom at least 50% were diagnosed at stage IV.^1^, ^50^ However, these studies were conducted in a period where breakthrough treatments were not available or in a province with inequities in public coverage of oral versus intravenous cancer drugs (e.g., Ontario).^54^

The inequalities we observed in the probability of receiving osimertinib as a second EGFR‐TKI were in the same direction as our survival inequalities. Given that, across material deprivation, we observed the largest variation in survival, but the smallest in receiving osimertinib, it is unlikely that longer survival solely explains the larger variations in osimertinib use observed across census‐based regions, immigration, and education. In our secondary analysis, patients residing in intermediary, remote, or northern health regions had a considerably lower likelihood of receiving osimertinib in comparison to university regions (Appendix D), confirming the possibility of confounding by calendar time in our main health region‐based analyses. Our results on the use of an EGFR‐TKI (i.e., osimertinib) across income are similar to a previous study in Ontario reporting a small positive relationship.^14^

The inequalities in delay from the time of biopsy to first‐line targeted treatment we observed were not in line with the survival inequalities. Montreal patients experienced longer delays but also longer survival than those from other census‐based regions, and delays by health center‐based regions were the largest despite lacking important survival differences. It is possible that there is a threshold effect and that the observed contrasts in treatment delays (e.g., 45 vs. 35 days) do not translate into meaningful survival differences. The overall median delay of 39 days we observed is in line with delays reported in other studies in Quebec: (1) a median of 36 days from biopsy to first targeted therapy^55^; (2) a median of 29 days from diagnosis to first palliative treatment,^56^ and; (3) ≤28 days from diagnosis to first targeted therapy for 66% of patients receiving it.^57^ In our study, all medians of treatment delay were above 30 days, with the highest being 45 days for peripheral regions. While our estimates are close to targets adopted by Quebec institutions of 28 and 42 days from diagnosis to first lung cancer treatment,^55^, ^56^, ^57^ the inter‐regional variation highlights room for improvement. Institutions in Canada have also demonstrated the achievability of delays as low as 7–15 days from the first visit involving a pathological diagnosis of advanced lung cancer to targeted treatment initiation.^58^, ^59^ Since the 25th, 50th, and 75th percentiles of treatment delay in peripheral health regions were consistently 10 days longer than those in university regions, we believe that the health center‐based gap in treatment delays is more likely due to health system factors than patients' disease severity.

One limitation of this study is the small sample size which resulted in reduced statistical power and widening the confidence intervals. However, this is inevitable since approximately 12% of advanced lung cancer patients receive EGFR‐TKIs in Quebec.^60^ Our study was restricted to patients with public drug insurance, who may be older than privately insured patients. This may induce a selection bias given that access to private insurance is driven by income and employment, which are impacted by health and age, factors that also affect survival. However, we assume a low proportion of privately insured patients in Quebec among all gefitinib users during the study period. Patients aged 65 and over represent 70% of all new lung cancer cases^32^, ^61^ and belong to a provincial‐level age group in which 90% of residents are registered for public drug coverage.^54^, ^62^ Our analysis also accounted for differences in age across the different income groups, which allowed us to partially control for potential selection bias pathways diluting the relationship between SES and survival. Even though Canada's universal public health care system does not include outpatient prescription drugs, Quebec has a universal drug insurance policy that requires all residents to have prescription drug coverage (residents without private coverage are covered by a public program). Furthermore, oral cancer drugs that are available on the public formulary are fully covered by the public program.^54^ It would be interesting to repeat our analyses with health administrative data from other provinces in Canada that have a low potential for selection bias due to drug insurance. For example, Alberta Manitoba, and Saskatchewan, offer universal public coverage and full reimbursement of cancer drugs that are available on the public formulary, and have only 12%–13% of all take‐home cancer drugs sales paid by private insurance.^63^ Lastly, we estimated associations that cannot be explained by differences in age and sex; however, future analyses should jointly account for potential confounders like immigration (or geographical location), comorbidities, smoking, ethnicity, and calendar time.

To our knowledge, this is the first study investigating SES‐, SDS‐, and geography‐based inequalities in survival and treatment in a cohort of advanced lung cancer patients in Canada, in the era of breakthrough therapies. Our analyses suggest that socioeconomic, sociodemographic, and geographic inequalities in survival and in care with EGFR‐TKIs exist among patients receiving gefitinib as first‐line palliative treatment. Given that the use of breakthrough therapies is expanding and that about half of all patients are diagnosed at an advanced stage, we expect to see survival inequalities over time, in a similar direction as in our study, in the advanced lung cancer population. Future studies on survival inequalities should therefore include this population in a causal framework to identify mediators that can be acted upon with policy and clinical interventions.

## AUTHOR CONTRIBUTIONS

**Samia Qureshi:** Conceptualization (lead); data curation (lead); formal analysis (lead); funding acquisition (lead); methodology (lead); validation (lead); visualization (lead); writing – original draft (lead); writing – review and editing (lead). **Gino Boily:** Methodology (equal); validation (equal); writing – review and editing (equal). **Jim Boulanger:** Methodology (equal); validation (equal); writing – review and editing (equal). **Élisabeth Pagé:** Resources (lead); supervision (supporting); writing – review and editing (equal). **Erin Strumpf:** Conceptualization (supporting); methodology (equal); supervision (lead); validation (equal); writing – review and editing (equal).

## FUNDING INFORMATION

This research was part of a doctoral Health System Impact Fellowship awarded to S.Q. The fellowship was co‐funded by the Canadian Institutes of Health Research (CIHR‐Institutes of Health Services and Policy Research and Cancer Research) and the Institut national d'excellence en santé et services sociaux (funding number HI9‐166408). E.S. is also a holder of McGill University's William Dawson Scholar award, which supports her research.

## CONFLICT OF INTEREST STATEMENT

The authors declare no other conflicts of interest.

## ETHICS APPROVAL

Ethics approval was also granted by McGill University's Faculty of Medicine and Health Sciences Institutional Review Board on March 22, 2022 (study number: A04‐M30‐22A).

## Figures and Tables

**FIGURE C1:**
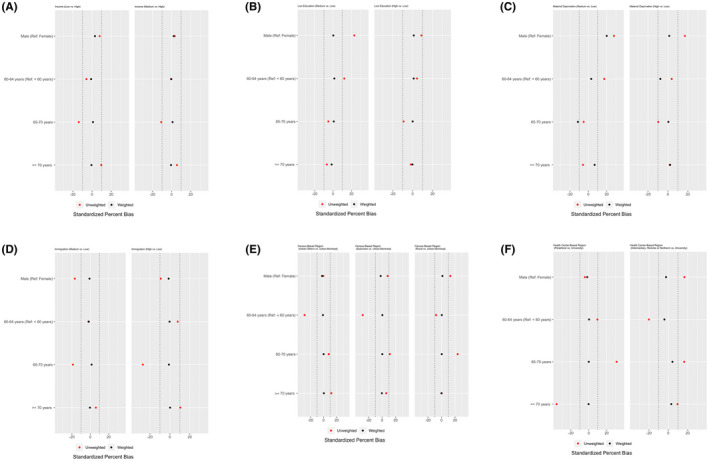
Balance in distributions of age and sex in cohort 1 before and after propensity score weighting, *N* = 457. (A) Income; (B) Low education; (C) Material deprivation; (D) Immigration; (E) Census‐based region; (F) Health center‐based region. $\text{Standardized percent bias}=100\times \left(\frac{{\hat{\rho }}_{\text{treatment}}-{\hat{\rho }}_{\text{control}}}{\sqrt{\frac{{\hat{\rho }}_{\text{treatment}}\left(1-{\hat{\rho }}_{\text{treatment}}\right)+{\hat{\;\rho }}_{\text{control}}\left(1-{\hat{\rho }}_{\text{control}}\right)}{2}}}\right)$, where ${\hat{\rho }}_{\text{treatment}}$ and ${\hat{\rho }}_{\text{control}}$ represent probabilities of the covariate in the comparison and reference groups, respectively, of a categorical treatment variable.

## Data Availability

The data that support the findings of this study are available on request from the Institut national d’excellence en santé et services sociaux (INESSS). The data are not publicly available due to privacy and ethical restrictions.

## APPENDIX A Intervention codes for medical interventions involving biopsies (Table A1).

**TABLE A1:** Biopsy‐related intervention codes in SMOD and MED‐ECHO.

(A) Physician billing codes for fee‐for‐service remuneration	
00181	PROCÉDÉS DIAGNOSTIQUES ET THÉRAPEUTIQUES; Biopsie (unique ou multiple); foie (à l'aiguille, percutanée) (PG‐28).
00184	PROCÉDÉS DIAGNOSTIQUES ET THÉRAPEUTIQUES; Biopsie (unique ou multiple); ganglion (cervical, axillaire ou inguinal) (PG 28).
00212	PROCÉDÉS DIAGNOSTIQUES ET THÉRAPEUTIQUES; Biopsie/cytologie à l'aiguille, par voie transcutanée, sous guidage échoscopique, fluoroscopique ou scanographique; osseuse; os.
00247	PROCÉDÉS DIAGNOSTIQUES ET THÉRAPEUTIQUES; Biopsie/cytologie à l'aiguille, par voie transcutanée, sous guidage échoscopique, fluoroscopique ou scanographique; osseuse; vertèbre.
00252	PROCÉDÉS DIAGNOSTIQUES ET THÉRAPEUTIQUES; Biopsie/cytologie à l'aiguille, par voie transcutanée, sous guidage échoscopique, fluoroscopique ou scanographique; rétropéritoine (rein, pancréas, ganglions, surrénale).
00273	PROCÉDÉS DIAGNOSTIQUES ET THÉRAPEUTIQUES; Biopsie (unique ou multiple); osseuse à l'aiguille (PG‐28).
00275	PROCÉDÉS DIAGNOSTIQUES ET THÉRAPEUTIQUES; RHINOSINUSOLOGIE; Thoracoscopie incluant, le cas échéant, biopsie, section d'adhérences et drainage, thoracique.
00308	PROCÉDÉS DIAGNOSTIQUES ET THÉRAPEUTIQUES; Biopsie (unique ou multiple); masse cervicale (à l'aiguille) (PG‐28).
00515	PROCÉDÉS DIAGNOSTIQUES ET THÉRAPEUTIQUES; Bronchoscopie rigide incluant la laryngoscopie, la trachéoscopie, la biopsie et l'exérèse de tumeur, le cas échéant; bronchoscopie rigide incluant la biopsie (PG‐23).
00524	PROCÉDÉS DIAGNOSTIQUES ET THÉRAPEUTIQUES; Bronchoscopie rigide incluant la laryngoscopie, la trachéoscopie, la biopsie et l'exérèse de tumeur, le cas échéant; médiastinoscopie avec ou sans biopsie.
00592	PROCÉDÉS DIAGNOSTIQUES ET THÉRAPEUTIQUES, Ponctions: (incluant injection s'il y a lieu); ganglion, un ou plusieurs.
00753	ACTES DIAGNOSTIQUES ET THÉRAPEUTIQUES; PNEUMOLOGIE; Bronchoscopie incluant la laryngoscopie, la trachéoscopie et la biopsie: localisation bronchoscopique d'un cancer occulte de l'arbre respiratoire, incluant biopsies multiples et aspirations cytologiques multiples, au niveau de toutes les bronches segmentaires.
00782	PROCÉDÉS DIAGNOSTIQUES ET THÉRAPEUTIQUES; Biopsie (unique ou multiple); par brossage bronchique (une ou plusieurs bronches) sans usage de bronchoscope ou laryngoscope, incluant l'intubation, l'anesthésie locale et la fluoroscopie.
00797	PROCÉDÉS DIAGNOSTIQUES ET THÉRAPEUTIQUES; Biopsie (unique ou multiple); plèvre ou poumon ou les deux: au trépan pneumatique.
02062	MUSCULO‐SQUELETTIQUE; SQUELETTE AXIAL; BASSIN; Biopsie osseuse; ouverte.
02066	MUSCULO‐SQUELETTIQUE; SQUELETTE AXIAL; THORAX; Biopsie; costale ouverte.
02109	MUSCULO‐SQUELETTIQUE; SQUELETTE AXIAL; COLONNE VERTÉBRALE; Biopsie; d'un élément postérieur.
02119	MUSCULO‐SQUELETTIQUE; SQUELETTE AXIAL; COLONNE VERTÉBRALE; Biopsie; corps vertébral.
02174	MUSCULO‐SQUELETTIQUE; EXTRÉMITÉS – MEMBRES SUPÉRIEUR; BRAS (HUMÉRUS); Biopsie osseuse; à l'aiguille.
02175	MUSCULO‐SQUELETTIQUE; EXTRÉMITÉS – MEMBRES SUPÉRIEUR; BRAS (HUMÉRUS); Biopsie osseuse; ouverte.
02247	MUSCULO‐SQUELETTIQUE; EXTRÉMITÉS – MEMBRES INFÉRIEURS; PIED; Biopsie; ouverte (PG‐28).
02719	MUSCULO‐SQUELETTIQUE; EXTRÉMITÉS – MEMBRES INFÉRIEURS; FÉMUR; Biopsie; forage et décompression de la tête fémorale.
02796	MUSCULO‐SQUELETTIQUE; EXTRÉMITÉS – MEMBRES INFÉRIEURS; FÉMUR; Biopsie; à l'aiguille.
02797	MUSCULO‐SQUELETTIQUE; EXTRÉMITÉS – MEMBRES INFÉRIEURS; FÉMUR; Biopsie; ouverte.
02864	MUSCULO‐SQUELETTIQUE; EXTRÉMITÉS – MEMBRES INFÉRIEURS; TIBIA ET PÉRONÉ; Biopsie; à l'aiguille.
02865	MUSCULO‐SQUELETTIQUE; EXTRÉMITÉS – MEMBRES INFÉRIEURS; TIBIA ET PÉRONÉ; Biopsie; ouverte.
02934	MUSCULO‐SQUELETTIQUE; EXTRÉMITÉS – MEMBRES SUPÉRIEUR; MAIN ET POIGNET; Biopsie; à l'aiguille, main et poignet.
02939	MUSCULO‐SQUELETTIQUE; EXTRÉMITÉS – MEMBRES SUPÉRIEUR; MAIN ET POIGNET; Biopsie; ouverte, main et poignet (PG‐28).
02991	MUSCULO‐SQUELETTIQUE; EXTRÉMITÉS – MEMBRES SUPÉRIEUR; AVANT‐BRAS; Biopsie – radius ou cubitus; à l'aiguille.
02992	MUSCULO‐SQUELETTIQUE; EXTRÉMITÉS – MEMBRES SUPÉRIEUR; AVANT‐BRAS; Biopsie – radius ou cubitus; ouverte.
03011	SYSTÈME RESPIRATOIRE; POUMONS ET PLÈVRE; Incision; Thoracoscopie diagnostique avec ou sans biopsie.
03013	SYSTÈME RESPIRATOIRE; POUMONS ET PLÈVRE; Incision; Thoracoscopie lors d'une autre intervention chirurgicale, supplément – NOTE: Le code 03013 ne peut s'ajouter à un acte comportant la mention « toute voie d'approche » sauf dans le cas d'une thoracoscopie suivie d'une thoracotomie.
03027	SYSTÈME RESPIRATOIRE; MÉDIASTIN; Incision; Médiastinotomie antérieure pour staging avec ouverture de la plèvre incluant la résection costale et le drainage, le cas échéant.
03035	SYSTÈME RESPIRATOIRE; MÉDIASTIN; Incision; Médiastinotomie pour exploration ou drainage: voie cervicale.
03036	SYSTÈME RESPIRATOIRE; MÉDIASTIN; Incision; Médiastinotomie pour exploration ou drainage: voie thoracique.
03120	SYSTÈME RESPIRATOIRE; POUMONS ET PLÈVRE; Incision; Thoracotomie; exploratrice avec biopsie.
03123	SYSTÈME RESPIRATOIRE; POUMONS ET PLÈVRE; Incision; Thoracotomie; exploratrice pour cancer, sans résection, avec ou sans biopsie.
04159	SYSTÈMES LYMPHATIQUE ET HÉMATOPOÏÉTIQUE; Excision; Exérèse de ganglions cervicaux (bénin ou malin) (PG‐28).
04161	SYSTÈMES LYMPHATIQUE ET HÉMATOPOÏÉTIQUE; Excision; Excision simple de ganglions lymphatiques pour lésion maligne (PG‐28).
07528	SYSTÈMES NERVEUX; CRÂNE & ENCÉPHALE; Lésions expansives tumorales: (incluant les lésions kystiques tumorales); Sus‐tentorielles: Biopsie diagnostique par trépanation seulement – (sans stéréotaxie, quel que soit le nombre de biopsies), par le neurochirurgien seulement.
07531	SYSTÈMES NERVEUX; CRÂNE & ENCÉPHALE; Lésions expansives tumorales: (incluant les lésions kystiques tumorales); Sous‐tentorielles: Tumeur du tronc cérébral; biopsie seulement.
07643	SYSTÈMES NERVEUX; CRÂNE & ENCÉPHALE; Installation du cadre; ponction ou biopsie (simple ou multiple) de lésion intraparenchymateuse, incluant la technique de localisation.
07690	SYSTÈMES NERVEUX; RACHIS, MOELLE, QUEUE DE CHEVAL; Tumorale; Ponction et/ou biopsie tumorale ou de kyste tumoral ou décompression.
08348	ULTRASONOGRAPHIE; ÉCHOGRAPHIE ARTICULAIRE; Échographie transendoscopique de l'oesophage, de l'estomac, du duodénum ou d'un organe intra‐abdominal incluant l'endoscopie gastro‐entérologique effectuée avec le scope d'échoendoscopie, maximum de un examen, par jour, par patient.
08349	ULTRASONOGRAPHIE; ÉCHOGRAPHIE ARTICULAIRE; Échographie transendoscopique de l'oesophage, de l'estomac, du duodénum ou d'un organe intra‐abdominal incluant l'endoscopie gastro‐entérologique effectuée avec le scope d'échoendoscopie; biopsie ou ponction ou injection, unique ou multiple, par voie transoesophagienne, transgastrique ou transduodénale d'une lésion médiastinale ou abdominale, supplément.
08383	ULTRASONOGRAPHIE; ÉCHOGRAPHIE DES VOIES RESPIRATOIRES; échographie endobroinchique.
08384	ULTRASONOGRAPHIE; ÉCHOGRAPHIE DES VOIES RESPIRATOIRES; échographie endobroinchique; avec ponction ganglionnaire ou tumorale transachéale ou transbronchique, supplément.
09367	PROCÉDÉS DIAGNOSTIQUES ET THÉRAPEUTIQUES; Bronchoscopie flexible incluant la laryngoscopie, la trachéoscopie, la biopsie, le lavage broncho‐alvéolaire et l'exérèse de tumeur, le cas échéant; Bronchoscopie: avec biopsie pulmonaire transbronchique, supplément.
09368	PROCÉDÉS DIAGNOSTIQUES ET THÉRAPEUTIQUES; Bronchoscopie flexible incluant la laryngoscopie, la trachéoscopie, la biopsie, le lavage broncho‐alvéolaire et l'exérèse de tumeur, le cas échéant; Bronchoscopie: avec ponction ganglionnaire transtrachéale et/ou transbronchique, supplément.
09369	PROCÉDÉS DIAGNOSTIQUES ET THÉRAPEUTIQUES; Bronchoscopie incluant la laryngoscopie, la trachéoscopie et la biopsie; bronchoscopie; avec lavage broncho‐alvéolaire diagnostique v.g. technique de crystal, supplément.
09418	PROCÉDÉS DIAGNOSTIQUES ET THÉRAPEUTIQUES; Ponctions (incluant injection s'il y a lieu); pleurale, sous guidage, le cas échéant (PG‐28).
09464	PROCÉDÉS DIAGNOSTIQUES ET THÉRAPEUTIQUES; Biopsie/cytologie à l'aiguille, par voie transcutanée, sous guidage échoscopique, fluoroscopique ou scanographique; thoracique.
09466	PROCÉDÉS DIAGNOSTIQUES ET THÉRAPEUTIQUES; Biopsie/cytologie à l'aiguille, par voie transcutanée, sous guidage échoscopique, fluoroscopique ou scanographique; hépatique.
09502	MUSCULO‐SQUELETTIQUE; EXTRÉMITÉS – MEMBRES INFÉRIEURS; PIED; Biopsie; à l'aiguille ou au trocart.
09550	MUSCULO‐SQUELETTIQUE; CRÁNE ET FACE; Biopsie; ouverte (unique ou multiple) (PG‐28).
20040	PROCÉDÉS DIAGNOSTIQUES ET THÉRAPEUTIQUES; Gastro‐entérologie; Endoscopie gastro‐entérologique: Oesophagoscopie rigide incluant la biopsie, le cas échéant (PG‐23).
(B) Canadian classification of health interventions (CCI) codes	
2AA71RW	Biopsie, méninges et duremère du cerveau, approche par trou de trépan avec aspiration à l'aiguille
2AA71SE	Biopsie, méninges et duremère du cerveau, approche par trou de trépan
2AA71SZ	Biopsie, méninges et duremère du cerveau, par craniotomie [volet osseux]
2AC71DA	Biopsie, ventricules cérébraux,approche endoscopique via un trou de trépan (ou la fontanelle)
2AE71DA	Biopsie, thalamus et noyaux gris centraux,approche endoscopique via un trou de trépan
2AE71SE	Biopsie, thalamus et noyaux gris centraux, accès par trou de trépan
2AE71SZ	Biopsie, thalamus et noyaux gris centraux, approche ouverte par craniotomie [volet osseux]
2AF71GR	Biopsie, région pituitaire, approche transluminale percutanée
2AF71QS	Biopsie, région pituitaire, approche transsphénoïdale ouverte [transethmoïdale]
2AG71SE	Biopsie, glande pinéale, approche par trou de trépan
2AJ71SE	Biopsie, cervelet, approche par trou de trépan (pour accéder au cervelet)
2AJ71SZ	Biopsie, cervelet, approche ouverte par craniotomie ou craniectomie [volet osseux]
2AN71SE	Biopsie, cerveau, approche par trou de trépan
2AN71SZ	Biopsie, cerveau, approche ouverte par craniotomie ou craniectomie [volet osseux]
2AP71SZ	Biopsie, tronc cérébral, approche ouverte par craniotomie ou craniectomie [volet osseux]
2AW71DA	Biopsie, moelle épinière, approche endoscopique (laparoscopie)
2AW71HA	Biopsie, moelle épinière,approche percutanée (à l'aiguille)
2AW71LA	Biopsie, moelle épinière,approche ouverte
2AX71DA	Biopsie, canal rachidien et méninges, approche endoscopique [laparoscopie]
2AX71HA	Biopsie, canal rachidien et méninges, approche percutanée
2AX71LA	Biopsie, canal rachidien et méninges, approche ouverte
2BG71DC	Biopsie, plexus brachial, approche endoscopique (thoracoscopie) par abord antérieur
2BG71LL	Biopsie, plexus brachial, approche ouverte antérieure
2EA71HA	Biopsie, crâne, approche percutanée (à l'aiguille)
2EA71LA	Biopsie, crâne, approche ouverte
2EB71LA	Biopsie, apophyse zygomatique,approche ouverte
2ED71LA	Biopsie, maxillaire,approche ouverte
2EE71LA	Biopsie, mandibule,approche ouverte
2GM70LA	Inspection, bronches,approche ouverte
2GM71BA	Biopsie, bronches,approche endoscopique par voie naturelle
2GM71BP	Biopsie, bronches approche endoscopique par voie naturelle, avec aspiration à l’ aiguille
2GM71BR	Biopsie, bronches, approche endoscopique par voie naturelle, brossage ou lavage
2GM71LA	Biopsie, bronches,approche ouverte
2GT70LA	Inspection, poumon NCA, approche ouverte
2GT71BA	Biopsie, poumon NCA, approche endoscopique par voie naturelle
2GT71BP	Biopsie, poumon NCA, approche endoscopique par voie naturelle et biopsie par aspiration
2GT71DA	Biopsie, poumon NCA, approche endoscopique [chirurgie thoracique vidéo assistée]
2GT71HA	Biopsie, poumon NCA, approche percutanée (à l'aiguille)
2GT71LA	Biopsie, poumon NCA, approche ouverte
2GW71DA	Biopsie, médiastin, approche endoscopique [chirurgie thoracique vidéo assistée]
2GW71HA	Biopsie, médiastin,approche percutanée (à l'aiguille)
2GW71LA	Biopsie, médiastin,approche ouverte
2GY70DA	Inspection, cavité thoracique NCA, approche endoscopique [chirurgie thoracique vidéo assistée]
2GY70LA	Inspection, cavité thoracique NCA, approche ouverte
2MA71HA	Biopsie, ganglion(s) lymphatique(s), région de la tête, approche percutanée (à l'aiguille)
2MA71LA	Biopsie, ganglion(s) lymphatique(s), région de la tête, approche ouverte
2MB71HA	Biopsie, ganglions lymphatiques de l'artère cervicale profonde,approche percutanée (à l'aiguille)
2MB71LA	Biopsie, ganglions lymphatiques de l'artère cervicale profonde,approche ouverte
2MC71HA	Biopsie, ganglion(s) lymphatique(s), cervical(aux), approche percutanée (à l'aiguille)
2MC71LA	Biopsie, ganglion(s) lymphatique(s), cervical(aux), approche ouverte
2MD71HA	Biopsie, ganglion(s) lymphatique(s), axillaire(s), approche percutanée (à l'aiguille)
2MD71LA	Biopsie, ganglion(s) lymphatique(s), axillaire(s), approche ouverte
2ME71BP	Biopsie, ganglion(s) lymphatique(s), médiastinal(aux), approche endoscopique par voie naturelle avec aspiration à l'aiguille
2ME71DA	Biopsie, ganglion(s) lymphatique(s), médiastinal(aux), approche endoscopique
2ME71HA	Biopsie, ganglion(s) lymphatique(s), médiastinal(aux), approche percutanée (à l'aiguille)
2ME71LA	Biopsie, ganglion(s) lymphatique(s), médiastinal(aux), approche ouverte
2MF71DA	Biopsie, ganglion(s) lymphatique(s), intrathoracique(s) NCA, approche endoscopique
2MF71HA	Biopsie, ganglion(s) lymphatique(s), intrathoracique(s) NCA, approche percutanée (à l'aiguille)
2MF71LA	Biopsie, ganglion(s) lymphatique(s), intrathoracique(s) NCA, approche ouverte
2MG71DA	Biopsie, ganglion(s) lymphatique(s), intraabdominal(aux), approche endoscopique
2MG71HA	Biopsie, ganglion(s) lymphatique(s), intraabdominal(aux), approche percutanée (à l'aiguille)
2MG71LA	Biopsie, ganglion(s) lymphatique(s), intraabdominal(aux), approche ouverte
2MH71DA	Biopsie, ganglion(s) lymphatique(s), pelvien(s), approche endoscopique
2MH71HA	Biopsie, ganglion(s) lymphatique(s), pelvien(s), approche percutanée (à l'aiguille)
2MH71LA	Biopsie, ganglion(s) lymphatique(s), pelvien(s), approche ouverte
2MJ71DA	Biopsie, ganglion(s) lymphatique(s), inguinal(aux), approche endoscopique
2MJ71HA	Biopsie, ganglion(s) lymphatique(s), inguinal(aux), approche percutanée (à l'aiguille)
2MJ71LA	Biopsie, ganglion(s) lymphatique(s), inguinal(aux), approche ouverte
2MK71HA	Biopsie, ganglion(s) lymphatique(s), membre(s) NCA, approche percutanée (à l'aiguille)
2MK71LA	Biopsie, ganglion(s) lymphatique(s), membre(s) NCA, approche ouverte
2MZ71HA	Biopsie, système lymphatique,approche percutanée (à l'aiguille)
2MZ71LA	Biopsie, système lymphatique,approche ouverte
2OA71DA	Biopsie, foie, approche endoscopique (laparoscopie)
2OA71GR	Biopsie, foie, approche veineuse transluminale percutanée
2OA71HA	Biopsie, foie,approche percutanée (à l'aiguille)
2OA71LA	Biopsie, foie,approche ouverte
2PB71DA	Biopsie, glande surrénale,approche endoscopique
2PB71HA	Biopsie, glande surrénale,approche percutanée (à l'aiguille)
2PB71LA	Biopsie, glande surrénale,approche ouverte
2SA71HA	Biopsie, atlas et axe,approche percutanée (à l'aiguille)
2SC71HA	Biopsie, vertèbres,approche percutanée (à l'aiguille)
2SC71LL	Biopsie, vertèbres, approche antérieure ouverte
2SC71PF	Biopsie, vertèbres, approche postérieure ouverte (postérolatérale)
2SE71HA	Biopsie, disque intervertébral, approche percutanée (à l'aiguille)
2SF71HA	Biopsie, sacrum et coccyx, approche percutanée (à l'aiguille)
2SF71LA	Biopsie, sacrum et coccyx, approche ouverte
2SK71HA	Biopsie, sternum,approche percutanée (à l'aiguille)
2SK71LA	Biopsie, sternum,approche ouverte
2SL71HA	Biopsie, côtes,approche percutanée (à l'aiguille)
2SL71LA	Biopsie, côtes,approche ouverte
2SM71HA	Biopsie, clavicule,approche percutanée (à l'aiguille)
2SM71LA	Biopsie, clavicule,approche ouverte
2SN71HA	Biopsie, omoplate,approche percutanée (à l'aiguille)
2SQ71HA	Biopsie, bassin,approche percutanée (à l'aiguille)
2SQ71LA	Biopsie, bassin,approche ouverte
2SW71HA	Biopsie, pubis,approche percutanée (à l'aiguille)
2SW71LA	Biopsie, pubis,approche ouverte
2TK71HA	Biopsie, humérus,approche percutanée (à l'aiguille)
2TK71LA	Biopsie, humérus,approche ouverte
2TV71LA	Biopsie, radius et cubitus,approche ouverte
2UJ71LA	Biopsie, phalanges de la main, approche ouverte
2VC71HA	Biopsie, fémur,approche percutanée (à l'aiguille)
2VC71LA	Biopsie, fémur,approche ouverte
2VP71HA	Biopsie, rotule,approche percutanée (à l'aiguille)
2VQ71HA	Biopsie, tibia et péroné,approche percutanée (à l'aiguille)
2VQ71LA	Biopsie, tibia et péroné,approche ouverte
2WL71LA	Biopsie, phalange du pied,approche ouverte
2WS71HA	Biopsie, métatarses,approche percutanée (à l'aiguille)
3GY30HJ	Ultrason, cavité thoracique NCA, par voie transoesophagienne
(C) Canadian classification of diagnostic, therapeutic, and surgical procedures (CCP) codes	
1682	Biopsie de la moelle et des méninges
2081	Biopsie percutanée (à l'aiguille) de la glande surrénale
2082	Autre biopsie de la glande surrénale
4581	Biopsie bronche/Biopsie des bronches par bronchoscopie
4582	Autre biopsie des bronches
4583	Biopsie percutanée (à l'aiguille) du poumon
4602	Thoracotomie d'exploration
4619	Médiastinotomie/Incision de médiastin
4681	Thoracoscopie/Thoracoscopie transpleurale
4682	Médiastinoscopie
4683	Biopsie de la paroi thoracique
4685	Biopsie percutanée (à l'aiguille) de médiastin
4686	Biopsie médiastine
4691	Thoracocentèse/Ponction pleurale
5281	Biopsie d'une formation lymphatique
6281	Biopsie percutanée du foie
6282	Autre biopsie due foie
8881	Biopsie d'os de la face
8990	Biopsie osseuse omoplate, calvicule, thorax, côtes, sternum
8991	Biopsie de l'os, humérus
8992	Biopsie osseuse radius, cubitus (avant‐bras)
8994	Biopsie osseuse fémur
8996	Biopsie osseuse tibia, péroné (cheville)
8998	Biopsie osseuse
8999	Biopsie osseuse siège autre ou non précisé
14811	Biopsie fermée [percutanée] [à l'aiguille] des méninges cérébrales
14821	Biopsie fermée [percutanée] [à l'aiguille] du cerveau
14822	Biopsie ouverte cérébroméningée

## APPENDIX B Details on exposures of interest (Table B1).

**TABLE B1:** Exposures of interest.

Name	Categories
Median income after tax^a^	Low: 0$–28,222$
	Medium: 28,223$–31,653$
	High: ≥31,654$
Percent with low education^a^ ^,^ ^b^	Low: 0%–28.69%
	Medium: 28.7%–34.99%
	High: ≥35.00%
Material deprivation index	Low: tercile 1 (least deprived)
	Medium: tercile 2
	High: tercile 3 (most deprived)
Percent immigrant^a^	Low: 0%–4.09%
	Medium: 4.10%–10.29%
	High: ≥10.30%
Census‐based region^c^	Urban (CMA): Montreal
	Urban (CMA): Others
	Suburban (CA)
	Rural (Non‐CMA/CA)
Health center‐based region	University
	Peripheral
	Intermediary
	Remote or Northern

Abbreviations: CA, census agglomeration; CMA, census metropolitan area.

^a^ The three categories were created using quintiles: quintiles 1 and 2 combined as the “Low” group, quintile 3 as the “Medium” group, and quintiles 4 and 5 combined as the “High” group.

^b^ Low education is defined as no secondary diploma or an equivalent attestation for individuals between 15 and 24 or ≥65 years, and no certificate or no post‐secondary education for individuals between 25 and 64 years.

^c^ The “CMA: Others” category includes the following cities: Quebec, Trois‐Rivières, Gatineau, Saguenay, and Sherbrooke.

## APPENDIX C Balance in distributions of age and sex before and after inverse probability weighting with propensity scores (Table C1 and Figure C1).

**TABLE C1:** Distributions of age and sex in cohort 1 before and after propensity score weighting, *N* = 457.

Propensity score weighting	Population	Age (years) *n* (%)				Sex *n* (%)	
		<60 years	60–64 years	65–70 years	≥70 years	Male	Female
Unweighted	Overall	58 (12.7)	48 (10.5)	102 (22.3)	249 (54.5)	144 (31.5)	313 (68.5)
Unweighted	Income^a^ ‐ Low	28 (14.0)	19 (9.5)	40 (20.0)	113 (56.5)	67 (33.5)	133 (66.5)
	Income^a^ – Medium	13 (13.1)	11 (11.1)	21 (21.2)	54 (54.5)	31 (31.3)	68 (68.7)
	Income^a^ ‐ High	17 (11.3)	17 (11.3)	39 (25.8)	78 (51.7)	45 (29.8)	106 (70.2)
	Low education^b^ ‐ Low	23 (11.1)	19 (9.2)	50 (24.3)	114 (55.3)	58 (28.2)	148 (71.8)
	Low education^b^ – Medium	10 (13.0)	10 (13.0)	17 (22.1)	40 (51.9)	30 (39.0)	47 (61.0)
	Low education^b^ – High	25 (14.6)	18 (10.5)	35 (20.5)	93 (54.4)	55 (32.2)	116 (67.8)
	Material deprivation^c^ – Low	18 (12.2)	13 (8.8)	37 (25.2)	79 (53.7)	37 (25.2)	110 (74.8)
	Material deprivation^c^ – Medium	15 (11.9)	18 (14.3)	29 (23.0)	64 (50.8)	48 (38.0)	78 (61.9)
	Material deprivation^c^ – High	22 (14.5)	15 (9.9)	32 (21.1)	83 (54.6)	50 (32.9)	102 (67.1)
	Immigration^d^ ‐ Low	15 (9.9)	14 (9.2)	45 (29.6)	78 (51.3)	53 (34.9)	99 (65.1)
	Immigration^d^ – Medium	11 (15.7)	6 (8.6)	15 (21.4)	38 (54.3)	19 (27.1)	51 (72.9)
	Immigration^d^ – High	32 (13.8)	27 (11.6)	42 (18.1)	131 (56.5)	71 (30.6)	161 (69.4)
	Census‐based region – Urban: Montreal	36 (14.1)	33 (13.0)	50 (19.7)	135 (53.1)	77 (30.3)	177 (69.7)
	Census‐based region – Urban: Others	11 (13.3)	4 (4.8)	19 (22.9)	49 (59.0)	25 (30.1)	58 (69.9)
	Census‐based region – Suburban	6 (14.6)	2 (4.9)	10 (24.4)	23 (56.1)	14 (34.1)	27 (65.9)
	Census‐based region – Rural	5 (6.5)	8 (10.4)	23 (29.9)	41 (53.2)	28 (36.4)	49 (63.6)
	Health center‐based region – University	31 (13.3)	24 (10.3)	40 (17.2)	138 (59.2)	72 (30.9)	161 (69.0)
	Health center‐based region – Peripheral	21 (14.8)	19 (13.4)	43 (30.3)	59 (41.5)	41 (28.9)	101 (71.1)
	Health center‐based region –Intermediary, remote or rorthern	6 (7.5)	4 (5.0)	19 (23.8)	51 (63.8)	31 (38.8)	49 (61.3)
Weighted	Income^a^ – Low	25.9 (13.0)	20.6 (10.3)	44.4 (22.2)	109.0 (54.5)	64,7 (32.4)	135.2 (67.6)
	Income^a^ – Medium	12.8 (12.9)	10.4 (10.5)	22.0 (22.2)	53.8 (54.5)	31.7 (32.0)	67.3 (68.0)
	Income^a^ – High	19.3 (12.8)	16.0 (10.6)	32.9 (21.8)	82.7 (54.8)	46.9 (31.0)	104.1 (69.0)
	Low education^b^ – Low	26.5 (12.9)	20.8 (10.1)	46.4 (22.5)	112.2 (54.5)	65.0 (31.6)	140.8 (68.4)
	Low education^b^ – Medium	10.0 (13.0)	8.1 (10.5)	17.5 (22.8)	41.3 (53.7)	24.4 (31.7)	52.5 (68.3)
	Low education^b^ – High	21.8 (12.8)	17.7 (10.4)	38.5 (22.5)	93.0 (54.4)	54.9 (32.1)	116.1 (67.9)
	Material deprivation^c^ – Low	18.3 (12.7)	16.5 (11.4)	35.1 (24.2)	75.0 (51.8)	42.1 (29.0)	102.9 (71.0)
	Material deprivation^c^ – Medium	16.6 (12.9)	16.0 (12.4)	25.1 (19.5)	71.0 (55.2)	49.4 (38.4)	79.3 (61.6)
	Material deprivation^c^ – High	20.6 (13.6)	13.7 (9.1)	37.0 (24.4)	80.2 (52.9)	44.8 (29.6)	106.7 (70.4)
	Immigration^d^ – Low	18.8 (12.4)	15.9 (10.5)	34.2 (22.6)	82.8 (54.6)	48.9 (32.2)	102.8 (67.8)
	Immigration^d^ – Medium	8.7 (12.4)	7.0 (10.0)	16.3 (23.2)	38.2 (54.4)	22.3 (31.8)	47.8 (68.2)
	Immigration^d^ – High	29.6 (12.4)	24.1 (10.4)	51.4 (22.2)	126.8 (54.7)	73.3 (31.6)	158.6 (68.4)
	Census‐based region – Urban: Montreal	32.4 (12.7)	26.3 (10.4)	57.0 (22.4)	138.4 (54.5)	80.2 (31.6)	173.8 (68.4)
	Census‐based region – Urban: Others	10.6 (12.8)	8.4 (10.1)	18.6 (22.4)	45.3 (54.7)	25.3 (30.5)	57.6 (69.5)
	Census‐based region – Suburban	5.2 (12.7)	4.3 (10.5)	9.2 (22.5)	22.3 (54.3)	12.5 (30.6)	28.5 (69.4)
	Census‐based region – Rural	9.7 (12.6)	8.1 (10.5)	17.3 (22.5)	41.8 (54.4)	24.8 (32.2)	52.1 (67.8)
	Health center‐based region – University	29.7 (12.7)	24.1 (10.3)	52.3 (22.4)	126.9 (54.5)	74.7 (32.1)	158.3 (67.9)
	Health center‐based region – Peripheral	18.1 (12.7)	14.8 (10.4)	31.8 (22.4)	77.3 (54.4)	44.5 (31.3)	97.4 (68.7)
	Health center‐based region –Intermediary, remote or northern	8.2 (10.6)	7.0 (9.1)	18.6 (24.2)	42.9 (56.0)	23.7 (31.0)	52.9 (69.0)

^a^ Low: 0–28,222$; Medium: 28,223$–31,653$; High: ≥31,654$.

^b^ Low: 0%–28.69%; Medium: 28.7%–34.99%; High: ≥35.0%.

^c^ Low: tercile 1; Medium: tercile 2; High: tercile 3.

^d^ Low: 0%–4.09%; Medium: 4.10%–10.29%; High: ≥10.30%.

## APPENDIX D Secondary analysis on likelihood of receiving osimertinib in cohort 1 (Table D1).

**TABLE D1:** Receiving osimertinib as a second EGFR‐TKI after receiving gefitinib.

	MainaAnalysis^a^ *N* = 457		Secondary analysis^a^ *N* = 195	
	Percentage (95% CI)	Percentage ratio (95% CI)	Percentage (95% CI)	Percentage ratio (95% CI)
SES and SDS variables				
Income				
Low: 0–28,222$	17.8 (12.5–22.6)	0.83 (0.53–1.29)	35.6 (25.8–47.8)	0.84 (0.56–1.30)
Medium: 28,223$–31,653$	22.2 (14.4–30.4)	1.03 (0.62–1.68)	48.8 (33.3–63.7)	1.15 (0.72–1.80)
High: ≥31,654$	21.5 (14.7–28.8)	REF	42.3 (30.3–53.5)	REF
Low education				
Low: 0%–28.69%	24.3 (18.6–29.5)	REF	47.0 (37.7–56.5)	REF
Medium: 28.7%–34.99%	15.8 (7.6–24.2)	0.65 (0.31–1.14)	36.8 (21.0–56.7)	0.78 (0.43–1.18)
High: ≥35.00%	16.9 (11.1–22.8)	0.69 (0.43–1.07)	34.8 (23.0–46.0)	0.74 (0.48–1.08)
Material deprivation index				
Low (least deprived)	21.7 (15.6–29.1)	REF	43.1 (30.6–54.6)	REF
Medium	24.2 (16.8–32.9)	1.12 (0.69–1.82)	45.1 (32.2–57.6)	1.05 (0.69–1.63)
High (most deprived)	16.3 (10.3–22.2)	0.75 (0.43–1.22)	32.8 (20.6–45.0)	0.76 (0.46–1.15)
Immigration				
Low: 0%–4.09%	12.7 (7.4–18.1)	REF	27.5 (17.0–38.4)	REF
Medium: 4.10%–10.29%	19.8 (11.0–28.8)	1.56 (0.75–3.03)	36.4 (20.0–54.1)	1.32 (0.67–2.51)
High: ≥10.30%	24.8 (19.4–29.9)	1.95 (1.26–3.36)	50.9 (40.8–61.5)	1.85 (1.22–3.13)
Geography variables				
Census‐based region				
Urban: Montreal	24.1 (18.5–29.6)	REF	47.1 (37.8–57.2)	REF
Urban: Others	9.4 (3.8–16.1)	0.39 (0.16–0.71)	26.6 (10.8–45.9)	0.56 (0.23–0.98)
Suburban	28.9 (14.7–44.5)	1.20 (0.60–2.05)	54.7 (33.7–77.7)	1.16 (0.70–1.75)
Rural	13.3 (5.9–22.1)	0.55 (0.25–0.98)	26.0 (10.9–41.7)	0.55 (0.24–0.90)
Health center‐based region				
University	19.8 (14.4–25.4)	REF	43.8 (34.1–54.6)	REF
Peripheral	22.7 (15.3–29.0)	1.15 (0.74–1.71)	44.3 (31.2–56.2)	1.01 (0.66–1.44)
Intermediary, remote or northern	16.1 (8.1–24.9)	0.81 (0.42–1.38)	28.8 (16.8–44.3)	0.66 (0.36–1.06)

Abbreviations: SDS, sociodemographic status; SES, socioeconomic status.

^a^ Analyses accounted for age (<60, 60–64, 65–70, ≥70 years) and sex.
